# Conversion of FeCo from soft to hard magnetic material by lattice engineering and nanopatterning

**DOI:** 10.1038/s41598-017-13602-x

**Published:** 2017-10-16

**Authors:** Takashi Hasegawa, Shunsuke Kanatani, Miyu Kazaana, Kairi Takahashi, Kohei Kumagai, Maiko Hirao, Shunji Ishio

**Affiliations:** Department of Materials Science, Akita University, 1-1 Tegata Gakuen-machi, Akita, 010-8502 Japan

## Abstract

The development of magnetic materials with large uniaxial magnetic anisotropy (*K*_u_) and high saturation magnetization has attracted much attention in various areas such as high-density magnetic storage, spintronic devices, and permanent magnets. Although FeCo alloys with the body-centred cubic structure exhibit the highest *M*_s_ among all transition metal alloys, their low *K*_u_ and coercivity (*H*_c_) make them unsuitable for these applications. However, recent first-principles calculations have predicted large *K*_u_ for the FeCo films with the body-centred tetragonal structure. In this work, we experimentally investigated the hard magnetic properties and magnetic domain structures of nanopatterned FeCo alloy thin films. As a result, a relatively large value of the perpendicular uniaxial magnetic anisotropy *K*_u_ = 2.1 × 10^6^ J·m^−3^ was obtained, while the *H*_c_ of the nanopatterned FeCo layers increased with decreasing dot pattern size. The maximum *H*_c_ measured in this study was 4.8 × 10^5^ A·m^−1^, and the corresponding value of *μ*_0_ *H*_c_ was 0.60 T, where *μ*_0_ represented the vacuum permeability.

## Introduction

The continuously increasing power consumption in motors^1^ and data storage devices^2,3^ containing permanent magnets has become a serious issue. In this regard, enhancing the performance of permanent magnets represents the simplest and most efficient method for reducing their power consumption. Since the energy utilized by permanent magnets depends on two parameters, coercivity (*H*_c_) and saturation magnetization (*M*_s_), high-performance motors must possess relatively high values of *H*_c_ and *M*_s_ to achieve sufficiently high flux densities. The ideal maximum energy product (*BH*)_max_ is defined by the following formula:

1 $${(BH)}_{{\rm{\max }}}={{M}_{{\rm{s}}}}^{2}/(4{\mu }_{0})$$

where *μ*_0_ is the vacuum permeability. This expression is based on the single domain theory that assumes hard magnetic properties of a material with a sufficiently high value of *H*_c_^4^. Among commercial permanent magnets^1^, FeNdB exhibits the highest (*BH*)_max_ of around 500 kJ·m^−3^, while relatively high uniaxial magnetic anisotropy (*K*_u_) is required for high-density magnetic storage, as illustrated by various bit-patterned media^2^. In order to prevent the generation of thermal fluctuations and, therefore, bit errors, the thermal stability factor *K*_u_ *V* of a magnetic layer must be much higher than the thermal energy *k*_B_ *T* (here *V* is the volume of a magnetic dot, *k*_B_ is the Boltzmann constant, and *T* is the temperature). The magnitude of *V* decreases with an increase in the storage density; hence, a relatively high *K*_u_ is required for maintaining high *K*_u_ *V*. A high *M*_s_ is also required for reading the magnetic flux from the recorded bits with low noise. By increasing the storage density, the number of hard disk drives utilized in data centres can be decreased, thereby reducing their power consumption.

Soft and hard magnetic materials are characterized by low and high *H*_c_ values, respectively. FeCo with the body-centred cubic (bcc) structure is a known soft magnetic material with the highest *M*_s_ among the currently studied transition metal alloys^5,6^. Although FeCo alloys exhibit relatively high *M*_s_, their low values of *K*_u_ and *H*_c_ make them unsuitable for the application as permanent magnets in motors and data storage. However, recent first-principles calculations have predicted a relatively high *K*_u_ for FeCo with the body-centred tetragonal (bct) structure^7–11^. The experimental *K*_u_ values have been also obtained for FeCo alloys with the bct structure^12–22^; they included a large *K*_u_ of over 10^6^ J·m^−3^ and lattice parameter ratio (*c*/*a*) between 1.15 and 1.25^22^. In addition, the *M*_s_ values that are equal to 85% of those measured for the bcc bulk Fe_50_Co_50_ have been reported as well^7,22^. However, the experimentally obtained *H*_c_ values were below 1% (10^3^ to 10^4^ A·m^−1^ ^12–22^) of the theoretical value.

The fabrication of dot patterns not only helps achieve high coercivity, determine a single domain size, and investigate the magnetization reversal process^21–25^; it is also essential for the development of bit-patterned media and innovative permanent magnets. A theoretical *H*_c_ value can be obtained when the dot volume is smaller than the size of a single domain after the coherent magnetization rotation according to the following expression derived from the single domain theory^4^:

2 $${H}_{{\rm{c}}}=2{K}_{{\rm{u}}}/{M}_{{\rm{s}}}$$

The *H*_c_ of dot patterns depends on the dot size and approaches the theoretical value in multilayer systems^23^. However, its magnitude for the ordered alloys is much lower than the theoretical value because of the non-uniformity of *K*_u_ and other physical parameters related to the non-uniformity of the order parameter inside the dots resulting from the annealing and ion milling processes^24^. The non-uniformity of the produced dot pattern can provide nucleation sites that induce magnetization reversal at a lower magnetic field than the theoretical *H*_c_ value. In that case, the demagnetizing field (*H*_d_) must be taken into account to obtain a high *H*_c_ value. The magnitude of *H*_d_ is defined by the following expression:

3 $${H}_{{\rm{d}}}=-N{M}_{{\rm{s}}}/{\mu }_{0}$$

where *N* is the demagnetizing factor, which depends only on the shape of the magnetic material^4^. The demagnetizing factors for the oblate spheroid having a major axis (*D*, *D*, *t*) with *k* = *D*/*t* > 1 are equal to

4 $${N}_{{\rm{D}}}=\,1/(2({k}^{2}\,-\,1))(({k}^{{\rm{2}}}\,\arccos (1/k)-1)/\sqrt{{k}^{2}\,-\,1})$$

5 $${N}_{{\rm{t}}}=1\,-\,2{N}_{{\rm{D}}}.$$

For a discoidal dot, the parameters *D* and *t* correspond to its diameter and thickness, respectively. A larger *t* results in a lower *H*_d_, which in turn produces a higher *H*_c_ according to the following expression:

6 $${H}_{{\rm{c}}}=p(2{K}_{{\rm{u}}}/{M}_{{\rm{s}}})\,-\,{H}_{{\rm{d}}}$$

where p is the factor related to the nucleation sites, which describes the non-uniformity of the physical parameters inside the dots (*p* < 1).

In this study, nanopatterned bct FeCo alloy layers were fabricated, and the effects of the dot size and FeCo layer thickness on their magnetic domain structure and *H*_c_ value, respectively, were investigated.

### Introduction of uniaxial magnetocrystalline anisotropy by lattice engineering

Prior to nanopatterning, the crystal structure and magnetic properties of FeCo/Rh/MgO (substrate) continuous films were evaluated. Accordingly, 10 at.% Al was added to the FeCo alloy, and the resulting ternary alloy is hereafter referred to as FeCo(Al). FeCo(Al) forms a CsCl-type (*B*2) ordered phase over a wide composition range^26^; hence, *K*_u_ was expected to be enhanced through *B*2 ordering and strong magnetoelastic interactions. Previous experiments have shown that the addition of 10 at.% Al results in the maximum value of *K*_u_^27^. The experimental composition of the alloy determined by electron probe X-ray microanalysis can be described by the formula (Fe_0.5_Co_0.5_)_90_Al_10_. The thickness of the Rh layer was fixed at 20.0 nm, while that of the FeCo(Al) layer *t* was varied between 2.0 and 20.0 nm. Rh was selected as a buffer layer material because it exhibited the lattice mismatch (*a*_FeCo_ − *a*_Rh_/$\sqrt{2}$)/*a*_FeCo_ ≈ 0.05, which was suitable for the introduction of epitaxial distortion into the FeCo(Al) structure. The [001] growth of FeCo on the Rh buffer surface characterized by the structural relation bct FeCo (001)[110] //Rh(001)[100] //MgO(001)[100] was confirmed by scanning transmission electron microscopy^22^. The order parameter estimated via X-ray diffraction (XRD) with synchrotron radiation was equal to 0.1–0.2.

**Figure 1:**
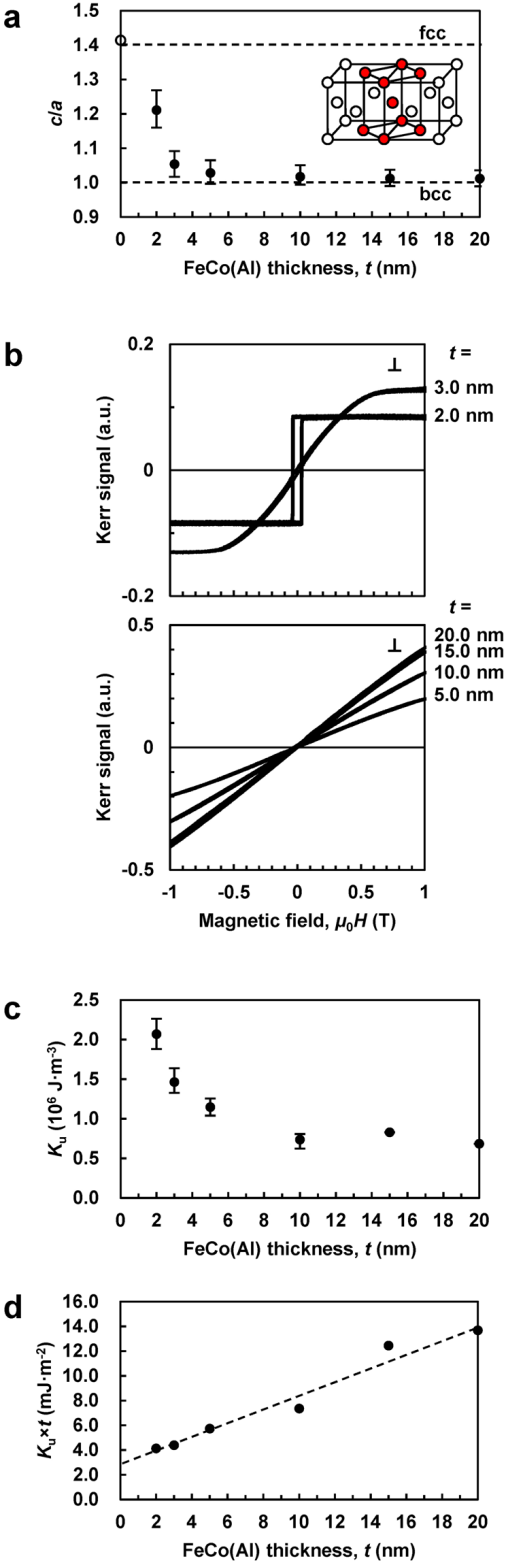
Crystal structure and magnetic properties of the continuous FeCo(Al) thin films. (**a**) *t* dependence of *c*/*a*. The error bars were calculated from the full widths at half maximum of the XRD peaks. Inset: illustration of the relationship between the fcc and bct unit cells at *c*/*a* = 1.41. The broken lines plotted at *c*/*a* = 1.00 and 1.41 correspond to the bcc and fcc structures, respectively. (**b**) Perpendicular magnetization curves (⊥). (**c**) *t* dependence of *K*_u_. The error bars were calculated from the noise widths of the magnetization curves recorded by the VSM. (**d**) *t* dependence of *K*_u_ × *t*.

The lattice parameter ratio *c*/*a* estimated for the bct unit cell of a FeCo(Al) layer (where *c* and *a* were evaluated via out-of-plane and in-plane XRD measurements) is plotted as a function of the film thickness (*t*) in Fig. 1a. The inset illustrates the relationship between the face-centred cubic (fcc) and bct crystallographic unit cells obtained at $c/a=\sqrt{2}\cong 1.41$. The *c*/*a* value at *t* = 0 is equal to 1.41, which is consistent with the value determined for the Rh fcc structure. The *c*/*a* value decreased with increasing *t*, reaching 1.01 at *t* = 20.0 nm because of the structural relaxation, and was slightly larger than that required for a bcc structure.

Figure 1b shows the perpendicular magnetization curves (⊥) obtained using the magneto-optic Kerr effect (MOKE) method, while Fig. 1c displays the *K*_u_ values plotted as a function of *t*. The *K*_u_ values were calculated via the following equation:

7 $${K}_{{\rm{u}}}={K}_{{\rm{u}}({\rm{e}}{\rm{f}}{\rm{f}})}+{{M}_{{\rm{s}}}}^{2}/(2{\mu }_{0})$$

where *K*_u(eff)_ is the effective magnetic anisotropy measured by a torque magnetometer. The *M*_s_ values were measured with a vibrating sample magnetometer (VSM). At *t* = 2.0 nm, the magnetic easy-axis was perpendicular to the film plane, and the magnitude of *K*_u_ was about 2.1 × 10^6^ J·m^−3^ with *M*_s_ = 1.95 Wb·m^−2^. At *t* ≥ 3.0 nm, the effective magnetic hard-axis was perpendicular to the film plane, and the value of *K*_u_ decreased with increasing *t*, reaching 6.8 × 10^5^ J·m^−3^ at *t* = 20.0 nm. The *K*_u_ value obtained at *t* ≥ 10.0 nm with *c*/*a* ≈ 1.01 can be attributed to the effect of *B*2 ordering (in addition to the tetragonal distortion)^9^.

Although the magnetization curves obtained for the samples with *t* ≥ 3.0 nm (Fig. 1b) correspond to the effective magnetic hard-axis perpendicular to the film plane, the magnetic easy-axis can be oriented perpendicularly to the film plane after correcting for the demagnetizing field characterized by the *μ*_0_ *H*_d_ value (equation (3)) of around 2.0 T. Figure 1d shows the *t* dependence of the product *K*_u_ × *t*. The intersection of the linear extrapolation of the obtained data with the vertical axis corresponds to the interfacial anisotropy (*K*_i_)^28^, yielding a value of around 2.8 mJ·m^−2^. Such a small value indicates that *K*_i_ is negligible in this system (in other words, the magnitude of *K*_u_ was entirely related to magnetocrystalline anisotropy). Recent first-principles calculations also indicated that the *K*_i_ estimated for the FeCo/Rh interface was relatively small^11^ and attributed the origin of the observed uniaxial magnetic anisotropy to the hybridization of the *d*_*xy*_ and *d*_*x*2−*y*2_ states of bct FeCo via spin-orbit interactions^9,10,22^. The [001] orientation of the bct FeCo(Al) films was perpendicular to the film plane; hence, uniaxial magnetocrystalline anisotropy was observed along the perpendicular direction due to spin-orbit interactions. The obtained data contradicts the results of previous studies, where the experimental *K*_u_ × *t*–*t* plot constructed for the CoFeB/MgO system exhibited a negative slope, indicating that the uniaxial magnetic anisotropy existed only at the CoFeB/MgO interface. Therefore, it can be concluded that the observed anisotropy was due to the hybridization between the CoFeB Fe-3*d* and MgO O-2*p* orbitals^29^.

### Conversion from soft to hard magnetic material by nanopatterning

**Figure 2:**
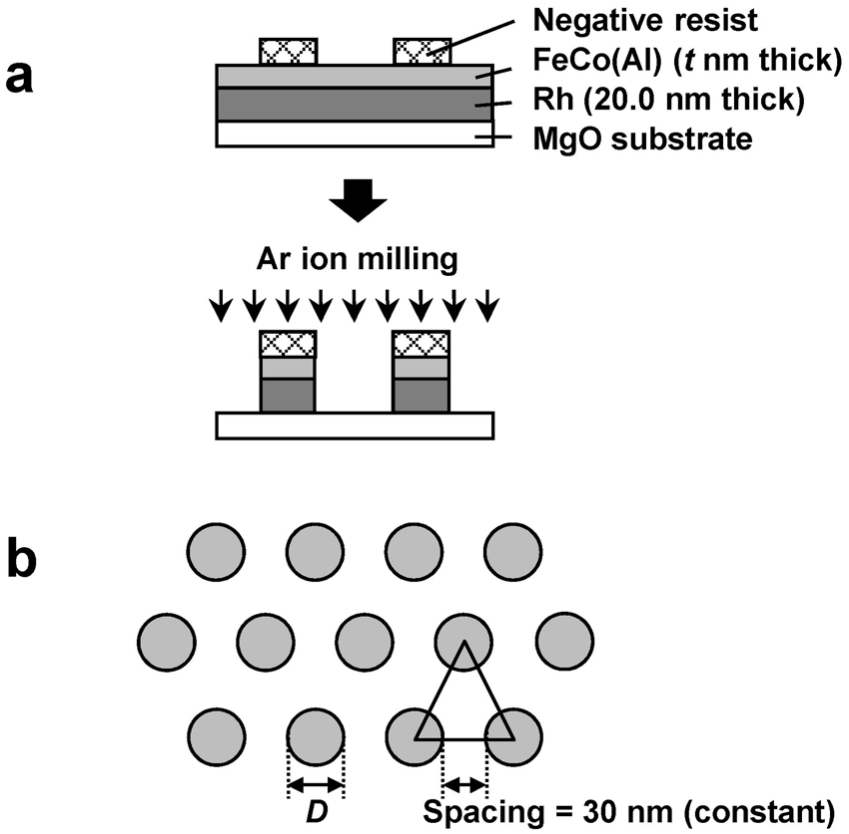
Nanopatterning process. (**a**) Fabrication via electron beam lithography. (**b**) A top view of the obtained dot patterns.

In order to evaluate the hard magnetic properties of FeCo(Al), circular dot patterns were fabricated using electron beam lithography and Ar ion milling techniques (Fig. 2a). Figure 2b shows the top view of the resulting dot pattern, where the diameter of the produced circular dots is denoted as *D*. The inter-dot spacing was 30 nm for all samples.

**Figure 3:**
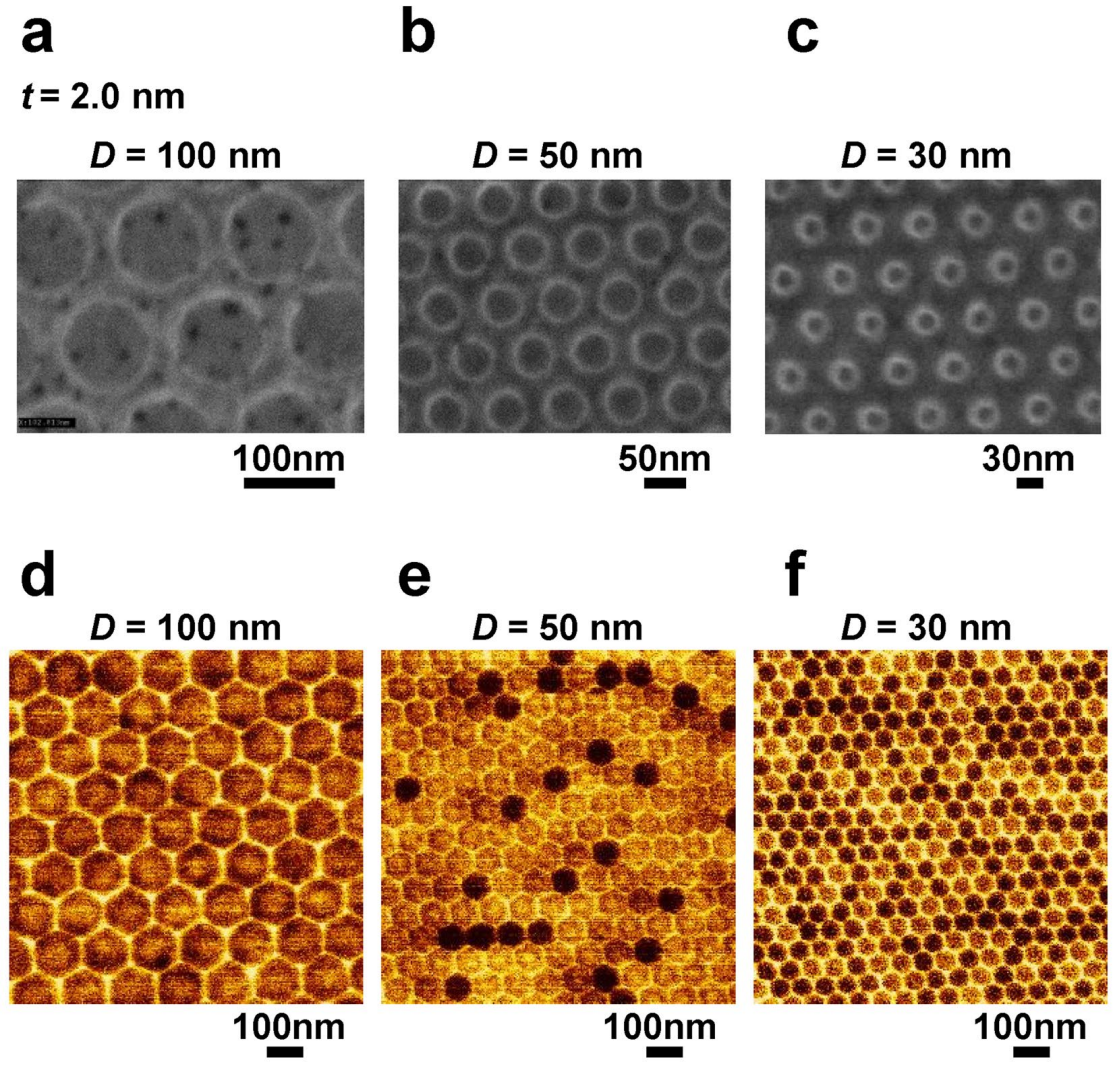
Surface topography and magnetic domains of the fabricated dots. (**a**–**c**) SEM images. (**d**–**f**) Demagnetized MFM images.

Figure 3a–f show the scanning electron microscopy (SEM) and demagnetized magnetic force microscopy (MFM) images of the patterned samples with *t* = 2.0 nm and *D* = 100, 50, and 30 nm, respectively. Before the MFM measurements, the samples were demagnetized by applying the in-plane magnetic field with a *μ*_0_ *H* of 1.8 T. The dots characterized by a multidomain structure, in which a bright core was surrounded by a dark ring, were observed at *D* = 100 and 50 nm (Fig. 3d and e). In contrast, dots with either a bright or dark contrast were observed at *D* = 30 nm (Fig. 3f), indicating that the single domain diameter in the remanent state was around 30 nm at *t* = 2.0 nm.

**Figure 4:**
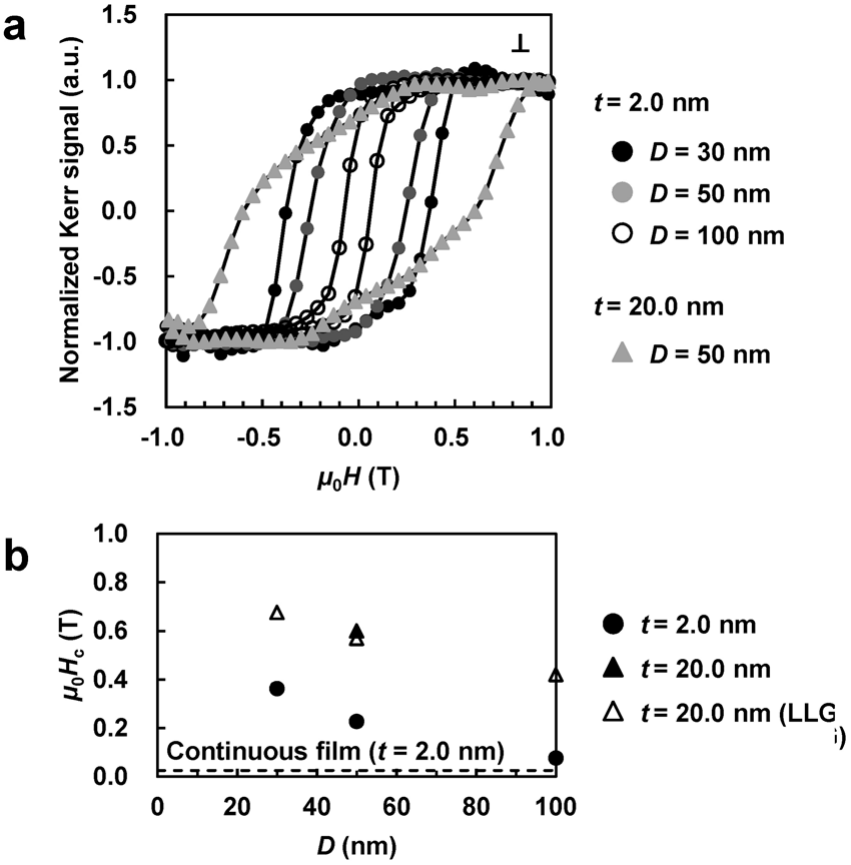
Magnetic properties of the nanopatterned samples. (**a**) Normalized perpendicular magnetization curves (⊥). (**b**) *D* dependence of *μ*_0_ *H*_c_ determined via experimental measurements (filled symbols) and theoretical simulations (open symbols).

Figure 4a shows the normalized perpendicular magnetization curves of the patterned samples recorded using the MOKE technique. The magnetic easy-axis of all the samples was perpendicular to the film plane; even the effective magnetic hard-axis was perpendicular to the plane of the continuous films with *t* ≥ 3.0 nm (Fig. 1b). The *N*_t_ values (equations (4) and (5)) obtained for the continuous films and dots with *t* = 20.0 nm and *D* = 50 nm (denoted by the triangles) were approximately 1.0 and 0.59, respectively. The reduction in *N*_t_ decreased *μ*_0_ *H*_d_ by 40% from 2.0 T to 1.2 T; hence, the magnetic easy-axis orientation was changed from the in-plane one to that perpendicular to the film plane because of the change in the aspect ratio *D*/*t*.

Figure 4b shows the *D* dependence of *μ*_0_ *H*_c_, which includes both the experimental results (the filled symbols) and data calculated via Landau–Lifschitz–Gilbert (LLG) micromagnetic simulations (the open symbols). The *K*_u_ values representing structural relaxation, which were plotted as functions of *t* (Fig. 1c) were used as the LLG parameters. The experimental *μ*_0_ *H*_c_ value obtained for the continuous film with *t* = 2.0 nm was equal to 0.025 T; its magnitude increased to 0.08, 0.23, and 0.36 T for the patterned samples with *D* = 100, 50, and 30 nm, respectively. The negative correlation between *μ*_0_ *H*_c_ and *D* was confirmed by conducting LLG simulations. Although a single domain was experimentally and theoretically observed for the dot with *t* = 2.0 nm and *D* = 30 nm (Fig. 3f), its experimental coercivity (0.36 T) did not reach the calculated *μ*_0_ *H*_c_ value of 1.4 T (not shown), indicating the existence of a high demagnetizing field and formation of nucleation sites represented by factor *p* (equation (6)).

The experimental *μ*_0_ *H*_c_ value obtained for the samples with *D* = 50 nm almost tripled after increasing *t* from 2.0 to 20.0 nm, which was likely due to the reduction of the demagnetizing field applied to the nucleation sites, whose *μ*_0_ *H*_d_ value decreased by 37% after increasing *t* from 2.0 to 20.0 nm at *D* = 50 nm. For instance, when *t* was increased to 50.0 nm at a constant value of *D* maintained at 50 nm (to ensure that *D*/*t* ≈ 1.0), the calculated *N*_t_ value decreased to approximately 0.33; consequently, the magnitude of *μ*_0_ *H*_d_ exhibited a 65% decrease with respect to the value obtained at *t* = 2.0 nm. Thus, the coercivity of FeCo(Al) increases with an increase in *t* and/or decrease in *D* until a single domain undergoes coherent rotation. Moreover, increasing the order parameter (via annealing) to reduce the number of nucleation sites and thus increase the magnitude of factor *p* (equation (6)) is also important for reaching high coercivity.

The maximum *μ*_0_ *H*_c_ of 0.60 T (corresponding to 68% of the theoretical value calculated using equation (2)) was obtained in this study for the nanopatterned sample with *t* = 20.0 nm and *D* = 50 nm. The experimental (*BH*)_max_ value of the nanopatterned sample calculated from the magnetization curve (depicted by the triangles in Fig. 4a) and *M*_s_ = 1.95 Wb·m^−2^ measured by the VSM is 150 kJ·m^−3^. Assuming that the single-domain dots exhibit coherent rotation with *M*_s_ = 1.95 Wb·m^−2^ and the total dot area on the two-dimensional surface equal to 90%, the ideal (*BH*)_max_ of this system calculated via equation (1) is expected to be around 687 kJ·m^−3^ or 140% of the value obtained for FeNdB.

## Conclusion

In summary, the tetragonally distorted FeCo(Al) thin film with a lattice parameter ratio *c*/*a* of 1.01–1.21 and high *K*_u_ of 2.1 × 10^6^ J·m^−3^ was fabricated. Further, a coercivity of 0.60 T was obtained for the nanopatterned FeCo layer with a thickness of 20.0 nm and dot pattern diameter of 50 nm. These results demonstrate that the combination of lattice engineering (introduction of tetragonal distortion into the structure of FeCo alloys) and nanopatterning (fabrication of patterns smaller than the single domain size) yields materials possessing high values of *K*_u_, *H*_c_, and *M*_s_. Therefore, the produced materials exhibit high potentials for future applications requiring high magnetic anisotropy and flux density, such as high-performance permanent magnets for motors and data storage devices.

## Methods

FeCo(Al) (*t* = 2.0−20.0 nm) films were prepared via dc-magnetron co-sputtering at a base pressure of 10^−7^ Pa and Ar gas pressure of 0.1 Pa. The composition of the produced films was controlled by varying the sputtering rates of the utilized Fe, Co, and Al targets and was determined using an electron probe X-ray microanalyser with an error of less than 1 at.% by averaging the compositions of 10 points on the surfaces of the film samples with dimensions of 1 cm × 1 cm. First, a 20.0 nm thick Rh buffer layer was grown on a single-crystalline MgO (100) substrate at 673 K. After decreasing the temperature to 473 K, FeCo(Al) films were prepared. Finally, a 2.0 nm thick SiO_2_ capping layer was sputtered onto the FeCo(Al) surface at 298 K. Calixarene-type resist (TEBN-1, Tokuyama Corp., Japan) was used during nanopatterning via electron beam lithography. The total area of the dot pattern was about 120 μm × 120 μm for each sample. The crystalline structure of the produced films was investigated via the out-of-plane and in-plane XRD with CuKα radiation. The degree of order parameter was measured by XRD with synchrotron radiation at a photon energy of 7.1 keV. The magnetization curves were recorded using the MOKE method under a magnetic field of up to 1.0 T applied perpendicularly to the film plane. The laser diameter of the MOKE measurement system was about 100 μm and significantly smaller than the patterned area of 120 μm × 120 μm. The *M*_s_ values were measured by the VSM with a magnetic field of up to 2.2 T. *K*_u(eff)_ was measured using a torque magnetometer with a magnetic field of up to 2.5 T. The sample surface topography and related magnetic domains were observed in vacuum by SEM and MFM, respectively. The cell size for the LLG simulations was 2 nm × 2 nm × 2 nm. The *t*-dependent *K*_u_ values (*K*_u_ = 2.1 × 10^6^, 1.5 × 10^6^, 1.1 × 10^6^, and 6.8 × 10^5^ J·m^−3^ for the regions with 0 ≤ *t* < 2.0 nm, 2.0 ≤ *t* < 4.0 nm, 4.0 ≤ *t* < 8.0 nm, and 8.0 ≤ *t* ≤ 20.0 nm, respectively) as well as *M*_s_ = 1.95 Wb·m^−2^ and the exchange stiffness constant of 1.8 × 10^−11^ J·m^−1^ were used as the LLG parameters.
